# Fine plastic foil as backing for sputtered nickel targets

**DOI:** 10.1007/s10967-013-2648-y

**Published:** 2013-08-02

**Authors:** Anna Stolarz, Raimo Seppälä

**Affiliations:** 1 Heavy Ion Laboratory, University of Warsaw, Ul. Pasteura 5a, 02-093 Warsaw, Poland; 2 Department of Physics, University of Jyväskylä, Survontie 9, P.O. Box 35 (YFL), 40014 Jyväskylä, Finland

**Keywords:** Nickel, Sputtering, Cu and C backing, Polyimide foil

## Abstract

Targets of ^58^Ni and ^60^Ni with areal density between 71 and 105 μg/cm^2^ backed with polyimide foil of 35–40 μg/cm^2^ were prepared by sputtering with Ar ions produced by a home made sputtering device at the Target Laboratory, University of Jyväskylä. The efficiency of the procedure was about 20 %.

## Introduction

Investigation of the distribution of the fusion-barrier height performed with ^20^Ne^9+^ ions provided by the Warsaw Cyclotron at Heavy Ion Laboratory, University of Warsaw required the targets of nickel isotopes: 58, 60 and 61 [1]. The required targets should be 80–100 μg/cm² thick with the target area of 15 mm in diameter and most preferably self-supporting although thin support by foils of light elements was acceptable as not disturbing the studied phenomena.

Preparation of self-supporting Ni targets in this thickness range is quite difficult. Although Ni layers of such thickness can be prepared in high vacuum by vapour deposition on substrate it is difficult to mount the produced foils to frame as Ni foils in this thickness range have a strong tendency to roll into tubes when self-supporting. This makes mounting the foils on the target frames when released from substrate very difficult if not impossible. To overcome this problem the thin Ni layer produced by vapour condensation or by sputtering, has to be, when still on the substrate, either additionally strengthened/stiffened by a support with low melting point removable under the beam or has to be produced on the backing which is removed after fixing the foils to the frame. Nevertheless, a method of producing Ni foils without the stresses responsible for this rolling effect has been recently reported in [2].

## Thin Ni layers production

### Sputtering device

Because of the nickel properties, i.e. its relatively high melting temperature and alloying with metals commonly used as the evaporation crucible nickel should be evaporated either by e-bombardment or e-gun heating or by sputtering.

Taking into account the minute amount of the Ni isotopes available for the target preparation and availability of the heating systems, the Ni foils were produced using the small home made sputtering device with sputtering voltage of 10 kV and Ar ions as sputtering projectiles available at the Target Laboratory of Physics Department, University of Jyväskylä.

The cathode applied in this device is made of graphite with the cavity of 5 mm in diameter for the sputtered substance which allows the target production with minute amount of the material. A short adjustable distance of the sputtered material and the substrate assures the high efficiency of the material collection. The deposit thickness inhomogeneity that may be caused by this short distance is reduced by the substrate rotation [3].

### Ni deposition

**Fig. 1:**
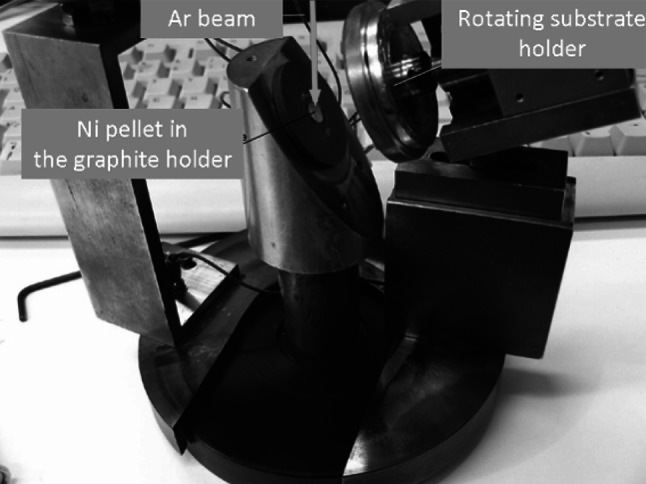
The heart of the sputterer; to the *left* is the graphite cathode with small cavity for the sputtered material; the rotating substrate holder is on the *right*

A pellet of compressed Ni powder was placed in the cavity of the graphite cathode (Fig. 1). The backing substrates were fixed to the rotating substrate holder placed at the distance of 2.3 cm from the Ni pellet. The thickness of the deposits was controlled by the time of sputtering process. The final thickness of the deposit was estimated using the weighing method. The targets were produced with the deposition rate varying between 1.3 and 2.5 μg/cm^2^/min.

#### On Cu substrate

**Fig. 2:**
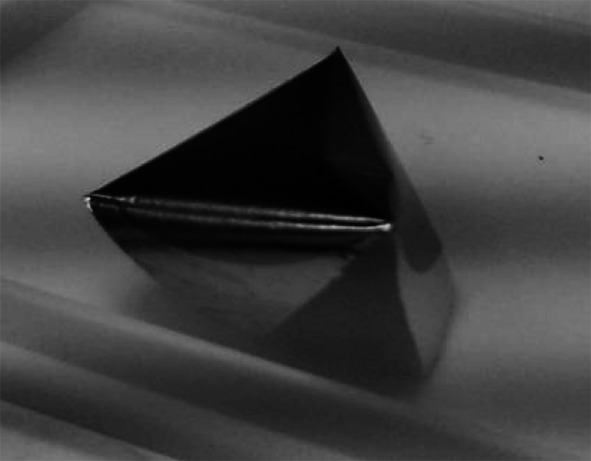
The nat Ni layer deposited on the 8 μm Cu foil

**Fig. 3:**
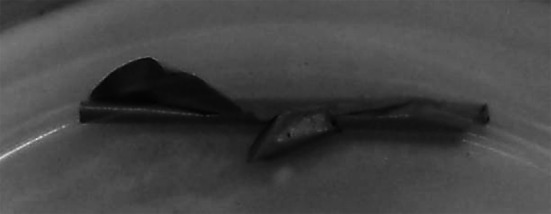
Ni foil made by sputtering on the Cu backing after etching the backing

The attempt to produce a set of Ni foils by the procedure described in [2] ended with no positive outcome. The Cu foil of 8 μm used as the backing was home prepared by rolling starting with Cu foils of 50 μm. Before applying as backing the Cu was annealed as recommended in [2] i.e. for ~5 min was heated in the vacuum at ~600 °C by resistant heating. The Ni layer deposited on the Cu backing built up with the stress sufficient to bend the Cu backing (Fig. 2), nevertheless the Ni was released from the backing by Cu etching with trichloroacetic acid and ammonia [2, 4]. The released foils were, in majority of cases, rolling itself into tubes (Fig. 3) making the mounting them on the frames infeasible.

**Fig. 4:**
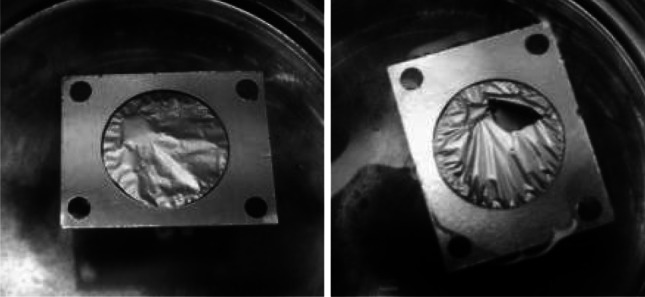
Ni foils deposited on the Cu backing during etching process

Fixing the foils to the frame before etching the copper backing as used for thicker Ni foils production by Arnison [4] gave no positive outcome either. The foils cracked either during the etching process (Fig. 4) or during the drying step.

The approach to the deposition of the sputtered Ni on substrate heated during Ni vapour deposition as recommended by Gursky [5] was not feasible in the device used for Ni layer production.

#### On low-*Z* backing

Taking into account no reproducibility of the foils production by deposition on the Cu backing further etched to obtain the self-supported foil and the fact that required targets were allowed to be backed by support of low-*Z* elements an attempt of depositing the Ni layers on a thin (10–12 μg/cm^2^) C foil was undertaken. It was not successful either. The C foil did not withstand bombardment by the sputtered Ni or the stress built up in the Ni foil during its deposition was too high. The frame after the sputtering was empty with only a few small scraps stuck to the substrate holder.

Finally targets for the experiment were prepared by nickel deposition on ~35 and 40 μg/cm^2^ polyimide foil (PIF) prepared in air or in argon [6]. The sputtering voltage applied in this process was ~6.5 kV.

**Fig. 5:**
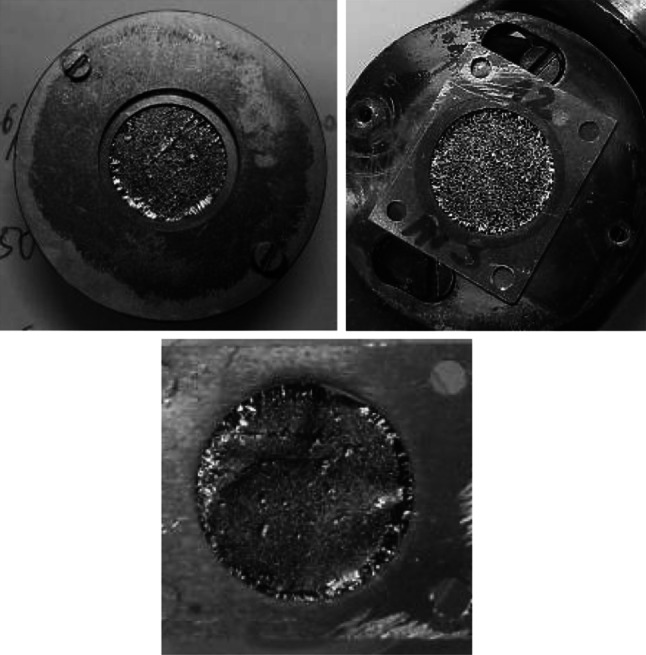
*Top left* the Ni target in the holder of the sputtering device (from the Ni side). *Top right* the *circle* of the Ni (*darker gray ring*) on the frame guarantee the contact with frame to facilitate the heat transfer from the target material. *Bottom* the view of the target from the PIF side

The PIF mounted to the frame was fixed to the rotating substrate holder using the mask, fixing the frame to the substrate and determining the size of the deposit (Fig. 5). Its aperture was 4 mm bigger than the aperture of the target frame. This was to assure a good contact of the Ni layer with target frame. It was aimed to enhance the dissipation of the heat deposited in the target by the bombarding beam.

The thickness of the deposit, as for deposition process on Cu was controlled by the time of sputtering process and the thickness of the completed deposit was estimated using the weighing method.

## Results

Applying described procedure the foils of natural nickel with areal density of 110–160 μg/cm^2^ and isotopic nickel, 58 and 60, with areal density of 71–115 μg/cm^2^ (Table 1) were produced with average material consumption amounting in ~2.5 mg per target.

**Table 1:** Isotopic targets prepared by Ni sputtering with 6.5 kV Ar ions

	Time (min]	Thickness (μg/cm^2^)	
Ni-58: target label			
PIF 4q	45		PIF - melted
PIF 7s	35	71.54	
PIF 8s	43.5	115.26	
PIF 9s	45	93.40	Slightly damaged on the side
PIF 10s	45	103.34	
PIF 11q	80	99.36	
Ni-60: target label			
PIF 11s	45	102.54	
PIF 12s	40	99.36	
PIF 5q	70	104.66	Small cut on the edge
PIF 8q	65.5	83.86	
PIF 9q (two runs)	70 + 20	105.33	

The behaviour of each PIF type under the nickel ‘projectiles’ initially differed but an additional thermal treatment (15 min at 350 °C in the oven) of PIFs made both types of the foil usable as backing for Ni deposition by the sputtering technique.

**Fig. 6:**
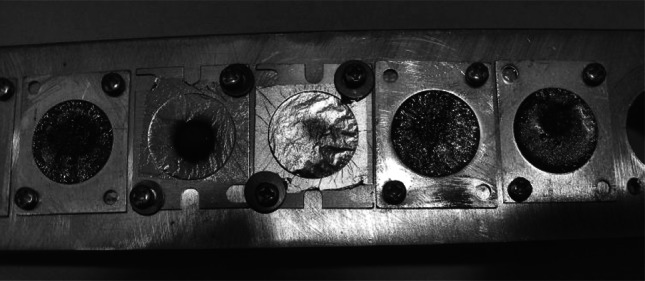
The set of Ni targets supported by PIF (from the *left* positions 1, 4, 5) and self-supporting (positions 2, 3) after bombardment by the ^20^Ne^9+^ beam of 50 MeV with 30 nA intensity

The thickness of a few randomly chosen targets was additionally determined with measurements of the alpha particles energy loss (Table 2). The obtained results confirmed the impact of the substrate rotation on the homogeneity of the deposit distribution (Fig. 6).

**Table 2:** Comparison of the thickness of the chosen Ni foils estimated by weighing and by α particles energy loss measurement

Targets	PIF μg/cm^2^ by α	Target thickness (μg/cm^2^)	
		Ni by weighing	By α
Ni 58 PIF 10s	40	103.34	135, 150, 154, 146
Ni 60 PIF 9q	35	105.33	146, 156

According to the report from the end users targets prepared by the method described here survived the bombardment by ^20^Ne^9+^ ions beam of ~50 MeV with intensity of 30 nA. It confirms the suitability of the PIFs as backings for targets used in the beam of heavy ions.
